# Dietary supplementation with *Bifidobacterium longum subsp. infantis (B. infantis)* in healthy breastfed infants: study protocol for a randomised controlled trial

**DOI:** 10.1186/s13063-016-1467-1

**Published:** 2016-07-22

**Authors:** Smita Awasthi, Reason Wilken, Forum Patel, J. Bruce German, David A. Mills, Carlito B. Lebrilla, Kyoungmi Kim, Samara L. Freeman, Jennifer T. Smilowitz, April W. Armstrong, Emanual Maverakis

**Affiliations:** Department of Dermatology, University of California Davis Medical Center, Sacramento, CA USA; Department of Food Science & Technology, University of California Davis , Davis, CA USA; Department of Chemistry, University of California Davis , Davis, CA USA; Division of Biostatistics, Department of Public Health Sciences, University of California Davis , Davis, CA USA; Foods for Health Institute, University of California Davis , Davis, CA USA; Department of Dermatology, Keck School of Medicine at the University of Southern California, Los Angeles, CA USA

**Keywords:** Bifidobacterium infantis, Probiotic, Atopy, Dose-escalation, Phase I clinical trial, Atopic dermatitis, Food allergy

## Abstract

**Background:**

The development of probiotics as therapies to cure or prevent disease lags far behind that of other investigational medications. Rigorously designed phase I clinical trials are nearly non-existent in the field of probiotic research, which is a contributing factor to this disparity. As a consequence, how to appropriately dose probiotics to study their efficacy is unknown. Herein we propose a novel phase I ascending dose trial of *Bifidobacterium longum subsp. infantis* (*B. infantis*) to identify the dose required to produce predominant gut colonisation in healthy breastfed infants at 6 weeks of age.

**Methods/design:**

This is a parallel-group, placebo-controlled, randomised, double-blind ascending dose phase I clinical trial of dietary supplementation with *B. infantis* in healthy breastfed infants. The objective is to determine the pharmacologically effective dose (ED) of *B. infantis* required to produce predominant (>50 %) gut colonisation in breastfed infants at 6 weeks of age. Successively enrolled infant groups will be randomised to receive two doses of either *B. infantis* or placebo on days 7 and 14 of life. Stool samples will be used to characterise the gut microbiota at increasing doses of *B. infantis*.

**Discussion:**

Probiotic supplementation has shown promising results for the treatment of a variety of ailments, but evidence-based dosing regimes are currently lacking. The ultimate goal of this trial is to establish a recommended starting dose of *B. infantis* for further efficacy-testing phase II trials designed to evaluate *B. infantis* for the prevention of atopic dermatitis and food allergies in at-risk children.

**Trial registration:**

Clinicaltrials.gov #NCT02286999, date of trial registration 23 October 2014.

## Background

The idea of probiotics, “live microorganisms that when administered in adequate amounts confer a health benefit on the host”, dates back to Elie Metchnikoff who hypothesised over 100 years ago that lactic acid bacilli had health benefits [1]. To date, thousands of reports of probiotics have supported the potential health benefits of these bacteria and the World Health Organization (WHO) has formulated guidelines for their use as nutritional supplements as well as investigation into their potential therapeutic properties [2]. Probiotics have been studied for the treatment and prevention of a variety of diseases in children including atopic dermatitis, bacterial gastroenteritis, inflammatory bowel disease, and necrotizing enterocolitis [3–5]. Overall, the data concerning probiotics as preventive agents for atopic diseases such as atopic dermatitis and food allergies are inconclusive, with some studies suggesting a possible benefit and others showing mixed results [4, 6–8].

Humans and other mammals are hosts to a diverse set of symbiotic as well as pathogenic intestinal bacteria. Approximately 1000 microbial species reside at a density of 1 × 10^12^ organisms per gram of colonic content [9, 10]. Several studies have demonstrated a clear link between immune system development and the composition of gut microbiota [11–13]. In addition, disruption of intestinal barrier function may lead to premature exposure of the infant to atopy-inducing environmental allergens, which would theoretically predispose the infant to the development of food allergies [14]. By protecting against colonisation by pathogenic bacteria, probiotics may help protect intestinal barrier function and thus decrease the susceptibility for development of atopic diseases and food allergies [15]. However, the pharmacokinetics of commensal bacteria and the dosage required to achieve predominant gut colonisation has thus far not been quantified. The probiotic that will be administered in our proposed phase I clinical trial, *Bifidobacterium longum subsp. infantis (B. infantis),* is unique in that it is able to fully utilise human milk oligosaccharides [16]. Thus, an exclusively breastfed infant’s gut provides the ideal environment to facilitate colonisation with this commensal bacteria [16–18]*.*

In order to proceed with designing a rigorous phase II clinical trial program to evaluate *B. infantis* as a preventative measure for a variety of childhood ailments, including atopic disease, it is necessary to first determine the pharmacologically effective dose (ED) of *B. infantis*. To date, pharmacologically guided phase I studies have not been conducted in the field of probiotics. We are thus proposing a phase I dose-escalation trial to evaluate the safety and pharmacokinetics of *B. infantis* supplementation when administered to healthy breastfed infants. The primary endpoint of the proposed trial is identification of the ED of *B. infantis*, defined as the dose required to produce predominant (>50 %) gastrointestinal colonisation in breastfed infants by 6 weeks of age. The 50 % value was chosen based on the proportion of *B. infantis* colonisation observed in breastfed infants in less-developed countries that have a low incidence of atopic disease compared to infants in the United States, specifically that seen in Bangladeshi infants [19, 20].

If successful, this study will be the first of its kind to establish a standardised dosing regime for probiotic supplementation in infants and will serve as a platform from which to design phase II and III trials to investigate the ability of *B. infantis* to protect against the development of a variety of childhood illnesses, including atopic dermatitis and food allergies.

## Methods/Design

### Study design

The proposed phase I clinical trial is a parallel-group, placebo-controlled, randomised, double-blind ascending dose study of dietary supplementation with *B. infantis* in healthy breastfed infants to evaluate its safety as well as determine the ED of *B. infantis* producing >50 % gut colonisation at 6 weeks of age. Infants will be enrolled sequentially in groups of five (three randomised to receive *B. infantis* and two to receive placebo). The trial participants and investigators will be blinded as to their group randomisation, which will be conducted by the study pharmacist (who will then dispense blinded *B. infantis* or placebo to the investigators). Depending on group assignment, each infant will receive one dose of either *B. infantis* or placebo on day 7 and another on day 14 of life (two doses total). For infants in the *B. infantis* group, a calculated maximally recommended starting dose (MRSD) will be used to initiate the dose escalation and is defined below. Every 2 weeks, an additional group of five infants (randomised 3:2 to *B. infantis* and placebo) will be enrolled to receive progressively higher doses of *B. infantis*. Calculation of the appropriate dose escalation will be performed using a modified Fibonacci series as described below in an effort to identify the ED of *B. infantis*.

**Fig. 1:**
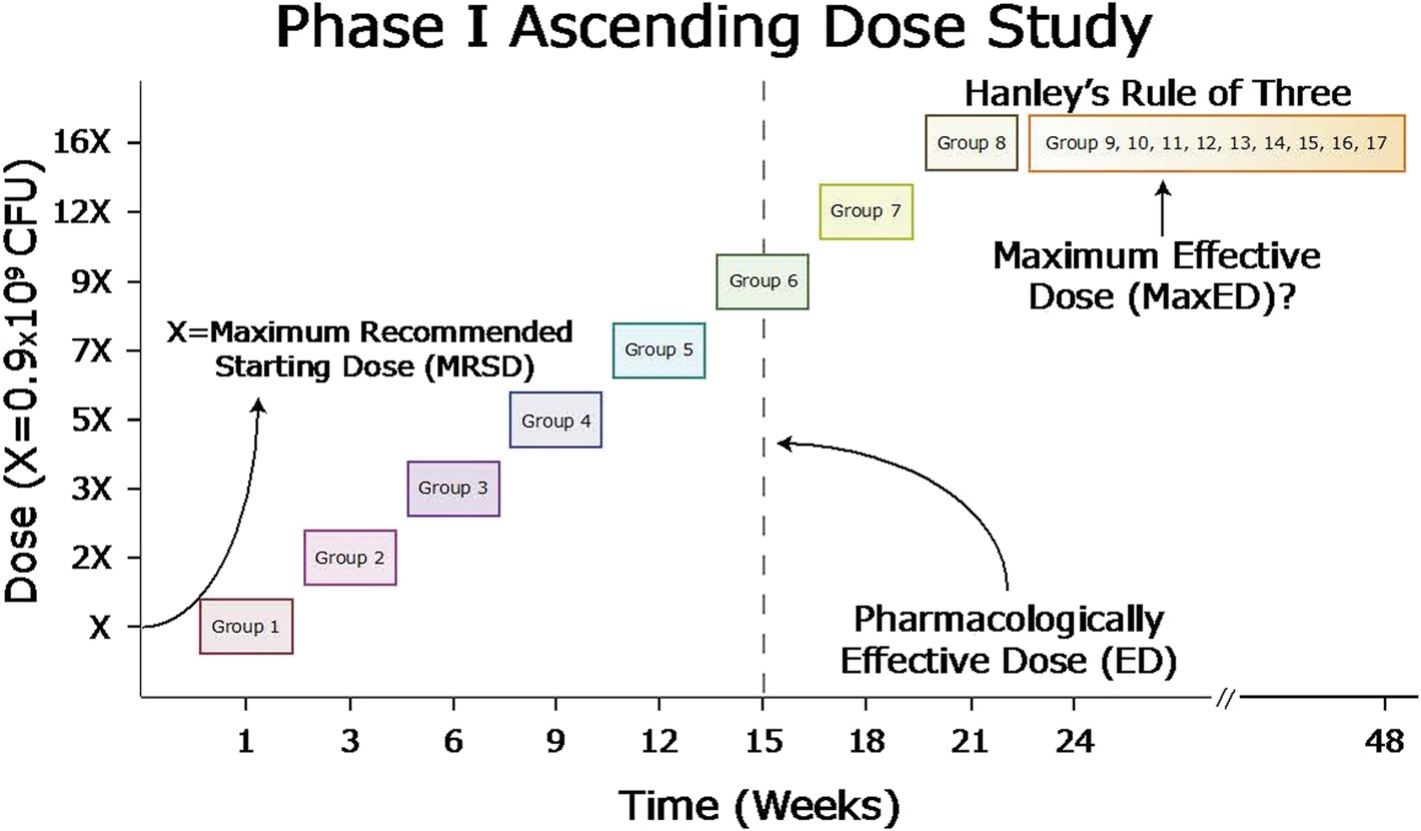
A parallel-group, placebo-controlled, randomised, double-blind ascending dose phase I study of dietary supplementation with *B. infantis.* Groups of healthy infants will receive increasing doses of *B. infantis* until it comprises 50 % of their gut microbiota, defined as the pharmacologically effective dose (*ED*). This figure arbitrarily designates Group 6 as receiving the ED of *B. infantis*. After the ED has been reached, two additional dose escalations will occur to determine the impact that additional *B. infantis* has on the gut microbiota. To satisfy Hanley’s Rule of Three, 30 infants will receive the highest dose of *B. infantis*. “*X*” represents the maximum recommended starting dose (*MRSD*). A modified Fibonacci Series (X, 2X, 3X, …) is used to guide the dose escalations. *CFU* colony-forming units

After the ED of *B. infantis* has been identified (defined as the dose capable of producing 50 % gut colonisation by 6 weeks of age) two additional sequential dose escalations will be performed. The purpose of the final two dose escalations is to determine if successively higher doses of *B. infantis* result in increased gut colonisation or barrier protection, or, alternatively, if a maximum effective dose (MaxED) for *B. infantis* exists above which there is no further increase in gut colonisation or barrier protection. Following the final dose escalation, Hanley’s Rule of Three will be applied in order to determine if lower-frequency adverse events are caused by *B. infantis*. Hanley’s Rule of Three states that in order to identify any adverse events occurring at a frequency of 1:10 or greater with a 95 % confidence interval, at least 30 subjects must be enrolled [21]. A schematic overview of the trial design and dose escalation protocol is provided in Fig. 1.

Study visits will be scheduled for weeks 1, 2, 6, 24, 36, 52, and 78. Parents will complete surveys at each study visit to monitor the infants for potential adverse events associated with probiotic administration including feeding intolerance, fevers, or bowel irregularities including constipation and diarrhoea.

Stool samples will be collected twice weekly for the first 6 weeks of life then once weekly at weeks 24, 36, 52, and 78. Stool samples will be analysed to determine the relative abundance of *B. infantis* over time, and the overall diversity of the gut microbiota with and without *B. infantis* supplementation. Stools will also be analysed for milk oligosaccharides to verify consumption of breast milk and to correlate the proportion of human milk oligosaccharides and free sugar monomers seen in the infant stool at various levels of *B. infantis* colonisation.

Breast milk will be collected at 7, 14, 42, 120, and 180 days after the birth of the infant to determine the type and proportion of milk oligosaccharides present at various time points. The purpose of this analysis is to allow correlation of the type and amount of milk glycans present in breast milk with that in the infant stool samples.

Entry into the study requires the intent to breastfeed exclusively for a minimum of 6 months. If mothers decide to discontinue breastfeeding during the study, we will note that in the infant’s notes and obtain an additional series of weekly stool samples for 6 weeks after discontinuation of breastfeeding. The purpose of this additional stool sample collection is to determine if discontinuation of breastfeeding has an impact on the level of existing *B. infantis* colonisation in the infant gut.

A table of the schedule of study visits and specimen collections is provided in Table 1, and an overview of the timeline of the study interventions and assessments is provided in Table 2.

**Table 1:** Schedule of study visits and specimen collections for each infant and mother

Week	Visits and activities
Prior to starting the study	• The patients’ parents or legal guardians will be asked to read and sign the consent form and will be instructed on the procedures for collection and storage of stool samples. They will be asked to refrain from giving the child any other dietary supplementation or probiotics while in the study and to inform the researchers if they are prescribed oral antibiotics at any time during the study.
Week 1	• Initial study visit, adverse event survey administration to parents and administration of first dose of *B. infantis* or placebo on day 7 of life.
	• Week 1 stool samples collected (infants will have stool samples collected twice weekly for the first 6 weeks and then at the study visits at weeks 6, 24, 36, 52, and 78).
	• If breastfeeding is discontinued at any point in the study, an additional series of six weekly stool samples will be collected following discontinuation in addition to the regularly scheduled collections
	• Mother collects breast milk on day 7 of life.
Week 2	• Study visit, adverse event survey administration to parents and administration of second dose of *B. infantis* or placebo on day 14 of life.
	• Week 2 stool samples collected
	• Mother collects breast milk on day 14 of life.
Week 3	• Week 3 stool samples collected
	• Adverse event survey administration to parents
Week 4	• Week 4 stool samples collected
	• Adverse event survey administration to parents
Week 5	• Week 5 stool samples collected
Week 6	• Study visit, adverse event survey administration to parents.
	• Week 6 stool sample collected.
	• Mother collects breast milk on day 42 of life.
Week 17	• Mother collects breast milk on day 120 of life.
Week 24	• Study visit, adverse event survey administration to parents.
	• Week 24 stool sample collected.
Week 25	• Mother collects breast milk on day 180 of life.
Week 36	• Study visit, adverse event survey administration to parents.
	• Week 36 stool sample collected.
Week 52	• Study visit, adverse event survey administration to parents.
	• Week 52 stool sample collected.
Week 78	• Study visit, adverse event survey administration to parents.
	• Week 78 stool sample collected.

**Table 2:** Schedules for study participant enrolment, interventions and assessments in parallel-group, placebo-controlled, randomised, double-blind ascending dose phase I study of dietary supplementation with *B. infantis*

	Enrolment	Allocation	Post-allocation											
Timepoint	–t_1_	0	Week 1	Week 2	Week 3	Week 4	Week 5	Week 6	Week 17	Week 24	Week 25	Week 36	Week 52	Week 78
Enrolment:														
Eligibility screen	X													
Informed consent	X													
Allocation		X												
Interventions:														
Dose #1 of *B. infantis*/placebo			X											
Dose #2 of *B. infantis*/placebo				X										
Infant stool collection			X	X	X	X	X	X		X		X	X	X
Breast milk collection			X (Day 7 of life)	X (Day 14 of life)				X (Day 42 of life)	X (Day 120 of life)		X (Day 180 of life)			
Assessments:														
Identification of pharmacologically effective dose (ED) of *B. infantis*								X						
Study visit			X	X				X		X		X	X	X
Adverse event survey			X	X				X		X		X	X	X
Analysis of stool oligosaccharides			X	X	X	X	X	X		X		X	X	X
Analysis of stool microbiota			X	X	X	X	X	X		X		X	X	X
Analysis of breast milk oligosaccharides			X (Day 7 of life)	X (Day 14 of life)				X (Day 42 of life)	X (Day 120 of life)		X (Day 180 of life)			

### Patient recruitment

As infants need to be enrolled within the first week of life, efforts will target pregnant women. Institutional Review Board (IRB)-approved flyers will be used to recruit pregnant women to participate in the study from the UC Davis Family Practice Center, UC Davis Obstetrics & Gynecology offices, and the inpatient Labor and Delivery ward.

### Informed consent process

During the initial screening visit, written informed consent will be obtained from the mother for infant participation in the study. The parents will be instructed on the proper collection and home storage of infant stool and maternal breast milk samples.

The potential risks and benefits to enrolled infants are outlined below in lay terms, as described in the IRB-approved consent forms that will be used to consent the parents/guardians of eligible infants for study participation:

“Probiotics are living organisms such as bacteria or yeast that are sold as dietary supplements for the purpose of improving health. Probiotics may have benefits such as decreasing the growth of harmful bacteria in the gastrointestinal tract, improving digestion and helping to strengthen the immune system. Probiotics have shown some benefit in reducing the risk of developing atopic (allergic) dermatitis in children. However, this optimal dose of probiotic needed to achieve such beneficial effects has not yet been studied in randomised controlled clinical trials. We wish to investigate the optimal dose of probiotic bacteria needed to colonise the infant gastrointestinal tracts by 6 weeks of age. We will also monitor patients for any evidence of atopic dermatitis (AD) and any effect on the severity of the AD during the course of the study.

Side effects described with probiotic use include diarrhea, vomiting and increased flatulence. Serious adverse effects of probiotics in infants include an extremely low risk of systemic infection (bacteraemia), but have thus far only been reported in children who were immunocompromised. Only healthy infants without any major systemic illnesses will be eligible to participate in this study, so we anticipate very minimal physical risks to subjects under these conditions.

However, as a result of being in the study your infant may experience one or more of the following adverse side effects listed below:

- Fever greater than 102°Farenheit
- Feeding difficulties (decreased feeding, colic, spitting up)
- Irregular bowel movements (diarrhea or constipation)
- Blood or pus in the stool
- Vomiting
- Abdominal pain or swelling
- Sepsis (serious infection involving the presence of bacteria in the baby’s blood)”

### Study inclusion and exclusion criteria

#### Inclusion criteria

- Healthy newborn infants between 1 and 7 days old with intent to be exclusively breastfed for a minimum of 6 months

#### Exclusion criteria

- Infants given dietary supplementation, including other probiotics.
- Infants born prior to 34 weeks gestation.
- Infants below 10^th^ percentile for body weight.
- Postnatal use of antibiotics (oral, intramuscular, or intravenous) by either the mother or the infant. Of note, prenatal maternal Group B streptococcus prophylaxis is not a criterion for study exclusion.
- Family history of immunodeficiency syndrome(s).
- Infants with signs of a clinically apparent underlying immunodeficiency.
- Intent to use non-breast milk infant formula for feeding during the first 6 months.
- History of gastrointestinal tract abnormality or infection.

### Protocol for calculating the starting dose of *B. infantis* and dose escalation

Previous studies of *B. infantis* supplementation in premature infants at the UC Davis neonatal intensive care unit (NICU) have used a dose of 1.4 × 10^9^ colony-forming units twice daily for 2 weeks without any observed serious adverse effects [22]. Applying a safety factor of three and adjusting for once-daily dosing yields, a MSRD of 0.9 × 10^9^ colony-forming units of *B. infantis* is to be administered on day 7 and day 14 of life. Each group of five infants will be enrolled at least 2 weeks apart to allow for sufficient time to identify possible adverse events prior to each dose increase. A modified Fibonacci-based dose escalation protocol will be applied as shown below:

$$ {F}_n={\displaystyle \sum_{k=0}^{\left[\frac{n-1}{2}\right]}}\left(\begin{array}{c}\hfill n-k-1\hfill \\ {}\hfill k\hfill \end{array}\right) $$

The specific modified-Fibonacci series will be as follows:

×, 2×, 3×, 5×, 9×, 12×, and 16× (where × = 0.9 × 10^9^ colony-forming units of *B. infantis*)

Based on the schedule for enrolling new groups every 2 weeks, by the time the pharmacologically effective dose (ED, defined as the dose of *B. infantis* resulting in 50 % gut colonisation at 6 weeks of age) has been identified, two additional dose escalations will have been performed in successive infant groups. Figure 1 provides schematic representation of infant group enrollment as well as the dose escalation protocol to identify the ED for *B. infantis* as well as the MaxED, if applicable.

### Study-wide number of subjects

We propose a maximum sample size of 90 infants. Five infants (three to receive *B. infantis* supplementation and two to receive placebo) will be enrolled for the initial dose of *B. infantis*. An additional five infants will be enrolled at each dose escalation. Once the pharmacologically effective dose (ED) of *B. infantis* has been reached, two additional dose escalations will be performed (for reasons as described above). We estimate that the ED of *B. infantis* will be identified within seven dose escalations (35 infants). Accounting for the final two dose escalations will bring the total to 45 infants. Following the final dose escalation, 45 additional infants (27 to receive *B. infantis* and 18 to receive placebo) will be enrolled to screen for lower-frequency adverse events using Hanley’s Rule of Three [21]. This will bring the maximum enrollment number to 90 infants. If the ED of *B. infantis* required to produce 50 % gut colonisation at 6 weeks is not reached, then enrollment will be halted after 90 infants have been enrolled. Additional stopping rules are listed in the “Study Endpoints” section.

### Randomisation

A computer-generated list of random numbers will be used to create a series of numbered, sealed opaque envelopes containing assignments to either placebo or supplementation with *B. infantis*. For every three infants assigned to *B. infantis* supplementation, two additional infants will be assigned to placebo. The study pharmacist will be responsible for the randomisation and delivery of the blinded supplements.

## Study methods and interventions

### Infant stool collection

Parents will be instructed how to collect and store infant stool samples at home. Stool samples will be collected twice weekly for the first 6 weeks of life then once weekly at weeks 24, 36, 52, and 78. If breastfeeding is discontinued at any point, an additional series of weekly stool samples will be collected for 6 weeks following discontinuation. At the initial enrollment visit parents will be provided with a stool sample collection including a sealable freezer box, marked sealed collection tubes and sealable plastic bags. Parents will be instructed to store samples in their home freezers immediately after collection to prevent secondary bacterial growth, and discard any samples not able to be frozen at the time of collection. Stool samples will be stored in the home freezers of study participants and picked up by study personnel once weekly to ensure they can be properly transported to the laboratory under temperature-controlled conditions. Any samples that are not stored immediately in the freezer at the time of collection or thaw en route to the laboratory will be discarded and parents will be instructed to collect and store a new stool sample according to the protocol. The stool samples will be analysed in the laboratory using DNA extraction to determine the identity and predominance of various commensal bacterial species, including the percentage of *B. infantis* present. The method of stool collection that will be provided to parents is described below:

1. At any time of day, scoop 1–2 teaspoons of your infant’s stool with the tongue depressor into the sealable collection tube. The amount of stool should fill the tube between the 5 and 10 ml mark.
2. Seal the tube and label with the time and date it was collected.
3. Place the stool filled tube inside a sealable plastic bag and seal it.
4. IMMEDIATELY place the sealed bag containing the stool sample into the study freezer box and store it in your freezer. It is important that stool samples are stored in the freezer immediately after collection to prevent bacteria from growing. If the stool sample was not immediately placed in the freezer, please discard the sample and collect another sample from your baby at the earliest convenience.

### Breast milk collections

Mothers will collect a series of breast milk samples for laboratory analysis to determine the type and proportion of milk oligosaccharides present at various time points. The purpose of this analysis is to allow correlation of the type and amount of milk glycans present in breast milk with that in the infant stool samples, both to verify the presence of the specific oligosaccharides preferred by *B. infantis* as well as to determine if appropriate amounts are present in the infant stool to represent exclusive breastfeeding practices.

Breast milk will be collected at 7, 14, 42, 120, and 180 days after the birth of the infant, and stored in the home freezers in the same manner as the stool samples. Parents will be provided with detailed instructions and supplies for collection as described below:

1. Between 2–4 hours after your last breastfeeding, use the breast pump to pump all the milk from one breast into the breast pump collection bottle.
2. Using the measuring cup, measure out 12 ounces of breast milk.
3. Divide the 12 ounces of breast milk into three (3) collection tubes, with four (4) ounces per tube.
4. Using the collection tube labels and a permanent marker, label each collection tube with the date and time of collection.
5. IMMEDIATELY, place the three tubes of breast milk in a single ziplock bag, seal it, and place in the study collection box stored in your freezer.
6. Wash and dry the breast pump, collection bottle, and measuring cup thoroughly after each use

### Data management and specimen banking

Collected breast milk and stool samples will be labelled with a four digit number randomly assigned to each patient as well as the date of sample collection. De-identified samples will be delivered to the laboratory for processing. Precautions will be taken to maintain the privacy of all participants. Personal information maintained on infant subjects will include age, first initial, last name, and four digit medical record number assigned at the time of randomisation. Patient information will not be disclosed to third party individuals except those authorised to oversee the research project.

## Study endpoints

### Primary outcome measures

#### *Identification of pharmacologically effective dose (ED) of* B. infantis

The primary endpoint of the study is identification of the ED of *B. infantis*, i.e. the dose required to produce predominant (>50 %) gut colonisation at 6 weeks of age. The value of 50 % colonisation was chosen because *B. infantis* represented greater than 50 % of the gut microbiota in the vast majority of Bangladesh infants [19]. The percent gut colonisation will be determined through analysis of stool samples by 16S sequencing.

#### Safety

An additional primary endpoint is to determine the safety of *B. infantis* supplementation in immunocompetent, full-term infants. Any adverse events including fever of 38.9 °C (102 °F) or higher, abdominal pain or colic, blood or purulence in the stool, diarrhoea or vomiting will be documented and dosing adjusted accordingly (“Stopping rules” are defined below).

### Secondary outcome measures

#### Milk oligosaccharide consumption

All stool samples will also be analyzed for the presence of oligosaccharides unique to breast milk and for the presence of free saccharide monomers, which are products of their incomplete digestion. *B. infantis* abundance will be correlated to these values.

#### Microbiota composition

In addition to determining the percent composition of *B. infantis* in infant stool samples, numerous other measures such as microbiota diversity (Shannon Diversity plots) and rate of *B. infantis* decline following cessation of breast feeding will also be determined.

### Provisions to monitor data and ensure safety of subjects

Children will be recruited for this study. Subjects will be monitored clinically during the study period to assess for potential adverse events. A previous study of *B. infantis* supplementation in premature infants administered similar doses and was well tolerated with no severe adverse events noted [22]. Nonetheless, patients enrolled in the study will be monitored closely and any evidence of feeding intolerance, illness, or infection will be thoroughly evaluated. A survey to assess for any baseline feeding intolerance or symptoms will be administered to all infants prior to administration of *B. infantis* or placebo. Additional symptom questionnaires will be administered weekly during the first month of the study and at every study visit therafter at 6, 24, 36, 52, and 78 weeks. Patients will be provided with the contact information for the principal investigator and on-call dermatology resident should any unforeseen symptoms or problems arise.

### Rules for stopping dose escalation and halting trial

1. Dose escalation will be stopped if a maximally tolerated dose (MTD) of *B. infantis* is reached. The data safety monitoring board will evaluate the adverse events reported at each ascending dose of *B. infantis*. If the safety monitoring board deems that one or more of the three infants in the *B. infantis* supplementation group experienced an adverse event (such as fever >38.9 °C (102 °F), abdominal pain or colic, blood or purulence in the stool, diarrhoea or vomiting) then additional dose escalation will be halted.
2. In the event dose escalation is halted due to adverse events, an additional group of five infants will be enrolled with three infants at the same (i.e. not escalated) dose of *B. infantis* and two receiving placebo.
3. If no adverse events occur in this additional, non-escalated group, then dose escalation will be resumed beginning with the next group of enrolled infants.
4. However, if adverse events are noted in one or more infant(s) in the non-escalated group, all further dose escalation will be halted. The *B. infantis* dose will be de-escalated to the previous value, and an additional group of five infants (three *B. infantis*, two placebo) will be enrolled. If no adverse events occur in the de-escalated group, this dose will be considered the MTD and the trial will be stopped.
5. If any adverse events are noted in the de-escalated group, further de-escalation and enrollment of infants will occur to identify the dose of *B. infantis* considered to be the MTD, in which no adverse events occur in the study group.

### Withdrawal of subjects

Subjects may be withdrawn without their consent if they acquire medical issues during the study period that require administration of oral or parenteral antibiotics or immunosuppressive medications. If a subject is to be withdrawn, the parent(s) will be contacted and the reason(s) for withdrawal will be explained in full. No further data will be collected from withdrawn subjects.

Subjects have the right to withdraw from the study at any time for any reason. Every effort will be made to follow-up subjects who discontinue placebo or *B. infantis* supplementation prior to the second dose. These evaluations should continue according to the protocol of scheduled study visits if at all possible. The reasons for discontinuation will be recorded in the subjects’ study file. If a subject is unable to return for evaluation, every effort will be made to contact via telephone 28 days after withdrawal to determine if any serious adverse events have occurred while off study. Any identified adverse events will be followed until resolution.

The investigator also has the right to remove subjects from the study without their consent. Possible reasons for removal include:

- Non-compliance with the study protocol
- Significant protocol deviation
- Serious adverse event potentially related to study treatment

Subjects that withdraw after the 6 week study visit will not be replaced with new subjects.

## Discussion

From our experience of administering *B. infantis* to infants in the NICU at UC Davis, it is highly unlikely that a phase I trial will successfully identify a MTD of this probiotic. Thus, our proposed phase I ascending dose study will instead utilise a targeted outcome, optimal gut colonisation of *B. infantis* at 6 weeks of age. Such “concentration controlled” phase I clinical trials that use pharmacokinetics in place of drug toxicity to guide dose escalation are supported in the literature [23, 24]. For example, in molecularly targeted anticancer agents, toxicity concerns are reduced, making “drug-related biological effects” a better suited primary endpoint [25–28]. Our specific trial will measure gut microbiota composition following *B. infantis* administration to determine the dose of *B. infantis* that is required to successfully colonise the gastrointestinal tract of a 6-week-old breastfed infant. Secondary outcome measures will include measurement of complex glycan consumption by the infant as well as diversity of the intestinal microbiota. As in all phase I studies, the infants will be monitored closely for adverse events. The main goal of this phase I trial is to establish a recommended administering dose of *B. infantis* to support further efficacy testing in phase II and phase III trials.

Prior probiotic studies have differed from the proposed study with regards to their dosing regime, inclusion criteria, and types of bacteria administered. Many prior trials in infants were designed with limited knowledge of the human milk glycome and resultant effects on the infant’s intestinal microbiotia. Probiotic bacteria used in prior trials in children have included *Bifidobacterium breve*, *Bifidobacterium lactis*, *Bifidobacterium longum*, *Lactobacillus acidophilus*, *Lactobacillus fermentum*, and *Propinobacterium freudenreichii*, among others [29–34]. In many prior studies, it was unclear why a particular probiotic was chosen. Research done by the milk group at UC Davis has shown that the vast majority of probiotic species do not grow on the human milk oligosaccharides in breast milk [16, 35, 36]. Evidently, most probiotics studied in previous clinical trials have been selected based on culturability and taste profiles when administered as fermented food products rather than their biological activity. In contrast, our proposed study is based upon research into the human milk glycome and insight into the specific bacteria that digest breast milk oligosaccharides.

One unique aspect of this trial is that we will administer *B. infantis* to exclusively breastfed infants. The breast milk will provide the prebiotic oligosaccharides and thus select for the growth of *B. infantis* over other gastrointestinal commensal bacteria. In addition, we will monitor the composition of the intestinal microbiota should the mothers choose to discontinue breastfeeding to determine the effect breast milk consumption has on the maintenance of *B. infantis* intestinal colonisation. Previous studies investigating probiotics have administered the bacteria in combination with galacto- and fructo-oligosaccharides [37, 38]. However, these sugars differ significantly from milk oligosaccharides in that they are linear rather than branched and lack fucose and sialic acid moieties [39]. As human breast milk will comprise the majority of an infant’s diet, supplementing with a probiotic that thrives on human milk oligosaccharides may be the most sensible strategy for cultivating a protective microbiota in healthy infants.

The most common adverse effects described with probiotic use include diarrhoea, vomiting, and increased flatulence. Probiotics are known to be extremely safe, as evidenced by prior use in premature infants as well as in both adults and children with HIV [40, 41]. In Finland, the use of probiotic supplements (namely *Lactobacillus rhamnosous GG*) has increased dramatically over the past 20 years without evidence of a corresponding increase in the rate of lactobacillus bactermia [42, 43]. Probiotic supplementation has also been shown to be well tolerated without adverse effects in a recent randomised, double-blind, placebo-controlled trial comparing a combination of lactobacilli (*Lactobacillus salivarius* and *Lactobacillus paracasei*) and bifidobacteria (*Bifidobacterium animalis subsp. lactis* and *Bifidobacterium bifidum*) in pregnant women and infants, with the goal of assessing the safety of probiotic supplementation [44]. In this trial, enrolled women were treated with a total of 1 × 10^9^ colony-forming units of this probiotic regime daily for the last month of pregnancy and the regime was then administered to their infants from birth through to 6 months of age. There was no significant difference in the incidence of adverse events in either the maternal or infant groups, and no adverse events were attributed directly to the probiotic supplementation. There have been rare reports demonstrating the possibility of probiotic-related infectious complications, such as sepsis, bacteraemia, and endocarditis [45–47]. However, it is estimated that the risk of developing bacteraemia from ingested *Lactobacillus* probiotics is less than one in one million [48]. Though extremely rare, sepsis due to bifidobacterium has been described [49–51]. The described cases of bifidobacterium bacteraemia include one adult that developed incidental sepsis following acupuncture, and two infants that received *Bifidobacterium breve* or *Bifidobacterium longum* as a probiotic supplement. Of note, one infant was premature with extremely low birthweight and the other was full term with comorbid omphalocele. To date there are no reported instances of bacteraemia resulting from *B. infantis*; however, all study participants will be warned of the extremely low risk of such complications.

While studies have demonstrated the ability of certain probiotics to prevent childhood illnesses [8], there are virtually no data on how to appropriately dose these supplements. The typical probiotic study has adopted a daily dosing regime, making feasibility an issue. Sometimes the probiotic is administered to the mother first and then to the infant for the first 6 months of life. Dose-ranging studies are usually required early on during a drug’s clinical development. Excluding a few exceptions, probiotic dose-ranging studies are for the most part non-existent [52–54]. As a follow-up clinical trial, we plan to conduct a dose-ranging phase II study that will compare the effectiveness of *B. infantis* daily dosing to a limited dosing regime consisting of *B. infantis* administered on days 7 and 14 only. In theory, the human breast milk oligosaccharides will bestow *B. infantis* with a competitive growth advantage, making daily dosing unnecessary. If the limited dosing regime is successful, it will have an immediate health care impact, as inoculating breast fed infants with two doses of *B. infantis* is an extremely feasible preventative measure to reduce the incidence of atopy and other diseases in at-risk children.

The pharmacokinetically guided design of this proposed phase I trial has not been attempted in prior probiotic studies. If successful the trial will identify a pharmacologically effective dose (ED) and maximum effective dose (MaxED) for *B. infantis* supplementation in healthy infants. The next generation sequencing strategy that will be employed to analyse the stool of the infants to determine gut colonisation with *B. infantis* has been well standardised, and is a common method for analysis of gut microbiota. Infants will also be monitored closely for potential adverse effects in case a maximally tolerated dose (MTD) is reached during the ascending dose study. The design of the trial is also innovative because the specific probiotic used for the supplementation will have a competitive advantage in an exclusively breastfed infant; it is the only bacterium that can fully utilise the complex oligosaccharides in breast milk as an energy source. Thus, the design of the trial provides an optimal environment for *B. infantis* to outcompete other intestinal microbiota. The rationale for the choice of probiotic used in prior infant trials is not clear; however, in this study it is based upon knowledge of the human milk glycome and specific metabolic needs of *B. infantis*. Our ultimate goal is to develop a clinically appropriate, safe, and effective dosing regime for probiotics that may be utilised in further phase II and phase III clinical trials. In doing so, we hope to lay the foundation for the design of objective, standardised clinical trials to assess the efficacy of probiotics for the prevention of a variety of diseases, including atopic dermatitis and food allergies in at-risk infants.

### Trial status

This protocol is for a proposed clinical trial. The phase I protocol has been reviewed and approved by the UC Davis Institutional Review Board (IRB). This trial has been registered through clinicaltrials.gov on 23 October 2014 and is accessible online (Identification Number NCT02286999). Patient recruitment has not yet commenced for this trial.

## Abbreviations

*B. infantis, Bifidobacterium longum subsp. infantis*; ED, pharmacologically effective dose*;* MaxED, maximum effective dose; MSRD, maximally recommended starting dose; MTD, maximally tolerated dose; NICU, neonatal intensive care unit
